# Construction of a high-density genetic map and QTL analysis for yield, yield components and agronomic traits in chickpea (*Cicer arietinum* L.)

**DOI:** 10.1371/journal.pone.0251669

**Published:** 2021-05-14

**Authors:** Rutwik Barmukh, Khela Ram Soren, Praveen Madugula, Priyanka Gangwar, P. S. Shanmugavadivel, Chellapilla Bharadwaj, Aravind K. Konda, Sushil K. Chaturvedi, Aditi Bhandari, Kritika Rajain, Narendra Pratap Singh, Manish Roorkiwal, Rajeev K. Varshney

**Affiliations:** 1 Center of Excellence in Genomics & Systems Biology, International Crops Research Institute for the Semi-Arid Tropics (ICRISAT), Hyderabad, India; 2 Department of Genetics, Osmania University, Hyderabad, India; 3 ICAR-Indian Institute of Pulses Research, Kanpur, UP, India; 4 Division of Genetics, Indian Agricultural Research Institute, Delhi, India; 5 Rani Lakshmi Bai Central Agricultural University, Jhansi, India; Mahatma Phule Krishi Vidyapeeth College of Agriculture, INDIA

## Abstract

Unravelling the genetic architecture underlying yield components and agronomic traits is important for enhancing crop productivity. Here, a recombinant inbred line (RIL) population, developed from ICC 4958 and DCP 92–3 cross, was used for constructing linkage map and QTL mapping analysis. The RIL population was genotyped using a high-throughput Axiom^®^*CicerSNP* array, which enabled the development of a high-density genetic map consisting of 3,818 SNP markers and spanning a distance of 1064.14 cM. Analysis of phenotyping data for yield, yield components and agronomic traits measured across three years together with genetic mapping data led to the identification of 10 major-effect QTLs and six minor-effect QTLs explaining up to 59.70% phenotypic variance. The major-effect QTLs identified for 100-seed weight, and plant height possessed key genes, such as C3HC4 RING finger protein, pentatricopeptide repeat (PPR) protein, sugar transporter, leucine zipper protein and NADH dehydrogenase, amongst others. The gene ontology studies highlighted the role of these genes in regulating seed weight and plant height in crop plants. The identified genomic regions for yield, yield components, and agronomic traits, and the closely linked markers will help advance genetics research and breeding programs in chickpea.

## Introduction

Chickpea (*Cicer arietinum* L.) is a major dietary grain legume cultivated widely in South Asia and the Middle East, with a genome size of ~740 Mb [1]. Globally, about 14.25 million metric tonnes of chickpea is produced annually on an area of 13.72 million hectares, and India with approximately 70% of the global production is the largest chickpea producer [2]. Chickpea is highly valued for its intrinsic potential for symbiotic nitrogen fixation and its dietary proteins, vitamins and essential minerals [3]. Despite its capability to produce 3.5–4.0 tonnes per hectare under optimum growing conditions, the global average chickpea productivity is approximately 1 tonne per hectare [4].

To ensure global food security and fulfil nutrient deficiency for 2 billion people worldwide in the coming decades [5], it is essential to increase chickpea production by developing high yielding varieties with consumer preferred traits. Genetic enhancement in yield components of chickpea is required to address the issues of low productivity. To develop improved high yielding chickpea varieties, it is crucial to understand the genetic basis of yield, yield components and associated agronomic traits [6]. Several attempts have been made to understand the genetic mechanisms associated with yield and its component traits in chickpea, and multiple QTLs have been identified for these traits. For instance, QTLs associated with plant height [7], flowering time [8], seed size/100-seed weight [9, 10], double poddedness [11], Fusarium wilt [12–14], Ascochyta blight [15, 16], *Helicoverpa armigera* resistance component traits [17], plant vigour [18], drought tolerance [4, 19], salinity tolerance [20–22] and heat tolerance [23] have been mapped in chickpea. Even though a large number of QTLs have been identified for multiple traits, very few have been deployed for marker-assisted selection (MAS) because QTLs for complex traits tend to have small effects and strong environmental influence. Further, they also need to be validated before being incorporated into MAS programs for chickpea genetic improvement.

Recent breakthroughs in genome sequencing technologies have reduced the cost of sequencing by several folds during the last decade [5]. This reduction in sequencing/genotyping cost has enabled the development of cost-effective low- to high-density genotyping platforms, resulting in the advancement of genomic resources for chickpea [24]. For instance, a high-throughput genotyping platform, ‘Axiom^®^*CicerSNP* array’, has facilitated high resolution genetic mapping by constructing high-density genetic maps to support genetics and breeding programs in chickpea [4, 20]. Such high-throughput genotyping platforms are very useful for trait mapping through genome-wide association mapping as well as for fine mapping of QTLs/trait in several crop species [25, 26].

The present study focused on constructing a high-density genetic map and identifying QTLs for yield, yield components and agronomic traits using a recombinant inbred line (RIL) population derived from ICC 4958 and DCP 92–3 cross. A dense genetic map developed in this study will help in the genetic dissection of other traits that differ between the two parental genotypes. Furthermore, potential genes underlying the marker intervals of the QTLs identified for 100-seed weight and plant height were retrieved. The QTLs and candidate genes detected in this study hold enormous potential to advance genetics research and breeding applications in chickpea.

## Results

### Phenotypic variation in the ICC 4958 × DCP 92–3 RIL population

The parental lines and RILs were analysed for their phenotypic performance across three years. Descriptive statistics, along with analysis of variance for six traits evaluated in the RIL population (ICC 4958 × DCP 92–3) across three years, have been provided in S1 Table. The mean squares and genetic parameters of the RILs showed highly significant (*p*<0.01 or *p*<0.001) variation for all the studied traits across three years (S1 Table). The highest coefficient of variation (%CV) was recorded for the number of primary branches (PB), followed by the number of secondary branches (SB) and the least for plant height (PLHT) (S1 Table). The magnitude of phenotypic coefficient of variation (PCV) was found to be higher than their corresponding environmental coefficient of variation (ECV) for yield per plant (YPP), 100-seed weight (100SW), pods per plant (PPP), and PLHT. The frequency distribution of the phenotypic data for the traits under study across three years indicated a marked variability and quantitative nature of the traits (S1–S3 Figs). Furthermore, the phenotypic trait value of many RILs surpassed their parental trait value in both directions for all the traits, except 100-seed weight, indicating the presence of transgressive segregation in this mapping population (S1 Table, S1–S3 Figs).

### Correlation among traits

To scrutinize the relationships between yield, yield components and agronomic traits evaluated across three years, a principal component analysis (PCA) was performed. For the experiment conducted during the Rabi season of 2015–16, the first two components explained ~59% of the total variability (S4 Fig). Here, YPP was closely related to PPP and PLHT. Similarly, 100SW and PB were found to be closely associated with PLHT and SB, respectively. For 2016–17 and 2017–18, the first two components explained about 60% and 71% of the total variation, respectively (S4 Fig). For both years, plant height favoured an increase in 100SW, while SB was closely related to YPP. Variation in the number of PB was tightly correlated with PPP. Furthermore, Pearson correlation analysis was performed to identify trait correlations within and between experimental trials (Table 1, S5 Fig). Here, 100SW displayed a significant and positive correlation with PLHT across all three years, viz. 2015–16 (r = 0.41, *p*<0.001), 2016–17 (r = 0.55, *p*<0.001) and 2017–18 (r = 0.47, *p*<0.001). A significant negative relationship was observed between 100SW and PPP for 2015–16 (r = -0.25, *p*<0.01) and 2017–18 (r = -0.19, *p*<0.05). A combined correlation analysis for all traits across three years was also undertaken to detect trait correlations (Table 1). The results indicated that, except for 100SW, all other traits displayed a moderate to low degree of correlation (ranging from r = ± 0.01 to r = ± 0.41) between years.

**Table 1:** Pearson correlation analysis for yield, yield components and agronomic traits evaluated across three years 2015–16, 2016–17 and 2017–18.

	PLHT_2015	PB_2015	SB_2015	PPP_2015	YPP_2015	100SW_2015	PLHT_2016	PB_2016	SB_2016	PPP_2016	YPP_2016	100SW_2016	PLHT_2017	PB_2017	SB_2017	PPP_2017	YPP_2017	100SW_2017
**PLHT_2015**	1.00																	
**PB_2015**	-0.07ns	1.00																
**SB_2015**	-0.16*	0.46***	1.00															
**PPP_2015**	0.08ns	0.23**	0.27***	1.00														
**YPP_2015**	0.23**	0.12ns	0.25**	0.53***	1.00													
**100SW_2015**	0.41***	-0.12ns	-0.06ns	-0.25**	0.14ns	1.00												
**PLHT_2016**	0.41***	-0.06ns	-0.08ns	-0.09ns	0.14ns	0.55***	1.00											
**PB_2016**	0.00ns	-0.08ns	0.00ns	-0.27***	0.16*	0.08ns	0.12ns	1.00										
**SB_2016**	0.20*	0.00ns	0.01ns	0.02ns	0.07ns	0.08ns	0.21**	0.37***	1.00									
**PPP_2016**	0.17*	0.05ns	0.08ns	0.13ns	-0.02ns	-0.01ns	0.18*	0.27***	0.48***	1.00								
**YPP_2016**	0.12ns	-0.09ns	0.03ns	-0.16*	-0.02ns	0.15ns	0.17*	0.24**	0.22**	0.38***	1.00							
**100SW_2016**	0.42***	-0.12ns	-0.07ns	-0.22**	0.17*	0.99***	0.55***	0.06ns	0.07ns	-0.02ns	0.14ns	1.00						
**PLHT_2017**	0.16*	-0.09ns	-0.07ns	-0.11ns	-0.08ns	0.31***	0.26***	0.04ns	0.05ns	0.10ns	0.07ns	0.30***	1.00					
**PB_2017**	0.04ns	0.10ns	0.04ns	0.03ns	-0.08ns	-0.13ns	-0.13ns	-0.04ns	0.03ns	0.03ns	-0.08ns	-0.13ns	0.15ns	1.00				
**SB_2017**	0.13ns	0.03ns	-0.12ns	0.04ns	-0.03ns	0.04ns	0.07ns	-0.09ns	0.13ns	0.06ns	-0.17*	0.03ns	0.33***	0.50***	1.00			
**PPP_2017**	0.07ns	0.05ns	-0.13ns	-0.04ns	-0.11ns	-0.13ns	-0.06ns	0.09ns	0.07ns	0.06ns	-0.12ns	-0.13ns	0.12ns	0.48***	0.55***	1.00		
**YPP_2017**	0.10ns	0.02ns	-0.13ns	-0.09ns	-0.11ns	0.12ns	0.04ns	0.14ns	0.08ns	0.01ns	-0.07ns	0.12ns	0.34***	0.37***	0.58***	0.79***	1.00	
**100SW_2017**	0.27***	-0.12ns	-0.08ns	-0.17*	-0.03ns	0.61***	0.37***	0.06ns	0.00ns	-0.02ns	0.10ns	0.60***	0.47***	-0.09ns	0.10ns	-0.19*	0.18*	1.00

***, ** and * indicate significant differences at *p*<0.001, *p*<0.01 and *p*<0.05, respectively; *ns*, non-significant.

### High-density genetic map

Out of the 50,590 SNPs available on the Axiom^®^*CicerSNP* array, a total of 17,173 SNPs were found polymorphic between two parents and displayed segregation within the mapping population. A genetic map was constructed using 3,818 SNPs that covered a distance of 1064.14 cM (Table 2), with a cumulative average distance of 0.30 cM between adjacent markers. The number of SNP markers mapped across eight linkage groups ranged from 126 (CaLG08) to 995 (CaLG04), spanning from 20.4 cM (CaLG08) to 230.4 cM (CaLG04) with a cumulative mean of 133.02 cM (Table 2). A large variation in marker density was observed across the linkage groups. The highest marker density of 6.16 SNPs/cM was observed on CaLG08, while CaLG03 displayed the lowest marker density of 1.80 SNPs/cM (Table 2).

**Table 2:** Summary of the genetic map constructed for ICC 4958 × DCP 92–3 RIL population using Axiom^®^*CicerSNP* array.

Linkage group	Number of SNPs mapped	Map distance (cM)	Inter-marker distance (cM)	Density (SNPs/cM)
CaLG01	560	122.00	0.22	4.59
CaLG02	532	139.90	0.26	3.80
CaLG03	351	194.50	0.55	1.80
CaLG04	995	230.43	0.23	4.32
CaLG05	249	112.98	0.45	2.20
CaLG06	491	103.84	0.21	4.73
CaLG07	514	140.04	0.27	3.67
CaLG08	126	20.45	0.16	6.16
**Total**	**3818**	**1064.14**	**0.30**	**3.91**

*SNPs*, single nucleotide polymorphisms*; cM*, centimorgan.

### QTL mapping for yield, yield components and agronomic traits

**Fig 1:**
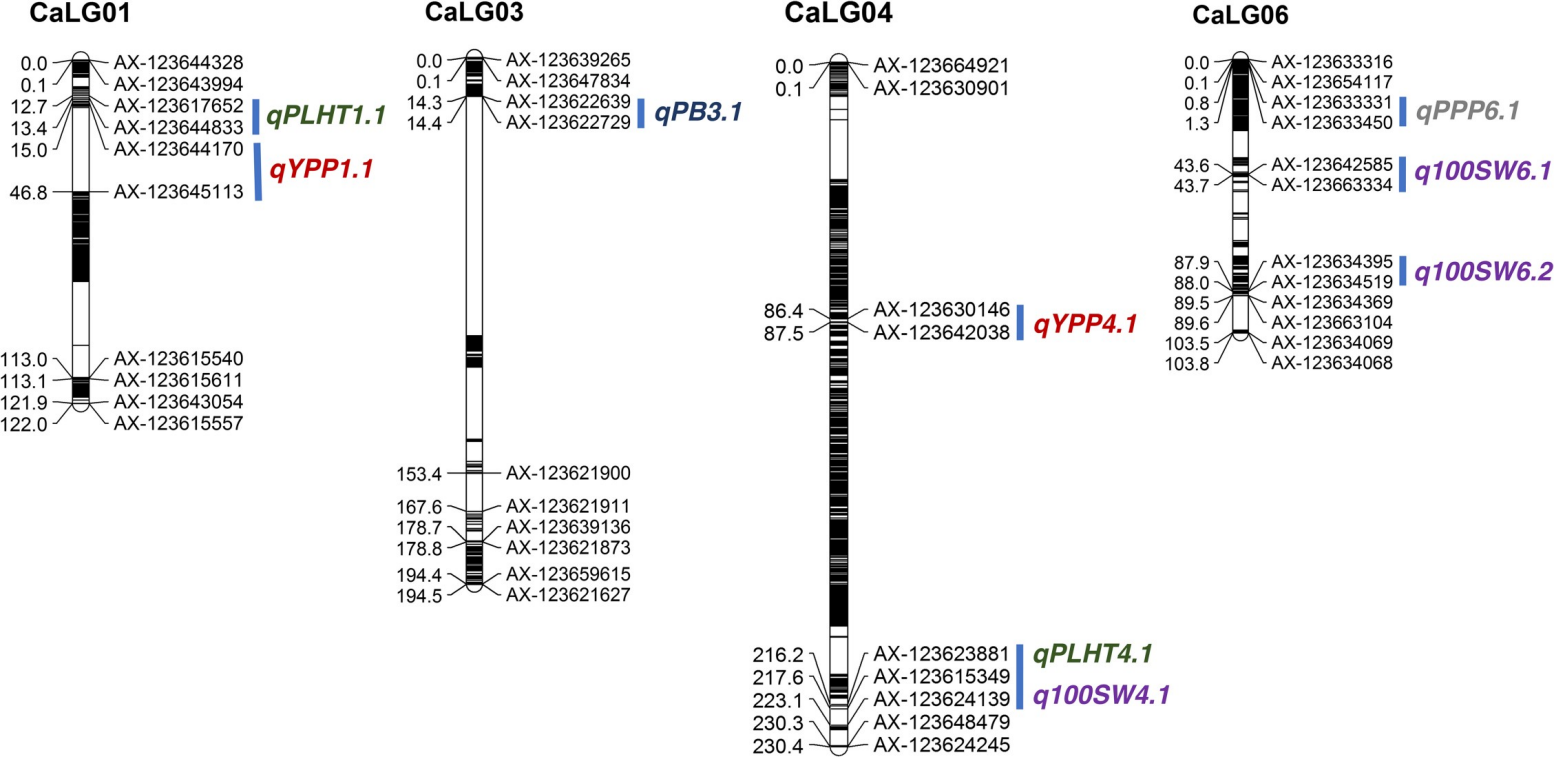
Graphical representation of linkage map with major-effect QTLs mapped for yield, yield components and agronomic traits in the ICC 4958 × DCP 92–3 RIL population. Major-effect QTLs for five traits are represented using their QTL names on the genetic map.

A total of 16 QTLs were identified for all studied traits, except for SB. These include 9 QTLs for yield and yield component traits (2 QTLs for YPP, 6 QTLs for 100SW, and 1 QTL for PPP) and 7 QTLs for agronomic traits (5 QTLs for PLHT, and 2 QTLs for PB) across three years. QTLs that contributed phenotypic variation explained (PVE) ≥ 10% were considered as major-effect QTLs, while QTLs with <10% PVE were considered as minor-effect QTLs. Based on these premises, a total of 10 major-effect QTLs were identified for five traits, viz., 2 QTLs for YPP, 3 QTLs for 100SW, 1 QTL for PPP, 3 QTLs for PLHT, and 1 QTL for PB (Fig 1). In addition, three minor-effect QTLs were identified for 100SW and three QTLs for agronomic traits (2 QTLs for PLHT, and 1 QTL for PB) across three years (Table 3).

**Table 3:** List of QTLs identified for yield, yield components and agronomic traits in ICC 4958 × DCP 92–3 RIL population.

Trait Name	QTL name	Year	Linkage group	Left marker	Right marker	Left marker position (cM)	Right marker position (cM)	LOD	PVE (R^2^%)	Additive effect
Yield per plant (YPP, g)	*qYPP4*.*1*	2015–16	CaLG04	AX-123642038	AX-123630146	86.44	87.52	3.50	10.10	-9.5
	*qYPP1*.*1*	2016–17	CaLG01	AX-123644170	AX-123645113	15.00	46.80	5.00	36.20	4.3
100-seed weight (100SW, g)	*q100SW6*.*1*	2015–16	CaLG06	AX-123642585	AX-123663334	43.66	43.70	5.70	13.00	4.0
	*q100SW7*.*1*	2015–16	CaLG07	AX-123637282	AX-123637281	47.61	47.77	3.60	8.10	1.7
	*q100SW3*.*1*	2016–17	CaLG03	AX-123621911	AX-123621900	153.40	167.60	3.30	6.50	2.3
	*q100SW6*.*2*	2016–17	CaLG06	AX-123634395	AX-123634519	87.91	88.02	5.20	11.30	3.6
	*q100SW7*.*2*	2016–17	CaLG07	AX-123635676	AX-123663528	139.78	140.04	3.70	7.80	1.7
	*q100SW4*.*1*	2017–18	CaLG04	AX-123615349	AX-123623881	216.23	223.07	6.90	16.60	2.6
Pods per plant (PPP)	*qPPP6*.*1*	2015–16	CaLG06	AX-123633331	AX-123633450	0.75	1.27	6.80	15.20	2.9
Plant height (PLHT, cm)	*qPLHT4*.*1*	2015–16	CaLG04	AX-123615349	AX-123623881	216.23	223.07	8.10	18.50	3.0
	*qPLHT1*.*1*	2016–17	CaLG01	AX-123617652	AX-123644833	12.70	13.40	6.20	14.20	3.2
	*qPLHT4*.*1*	2016–17	CaLG04	AX-123615349	AX-123623881	216.23	223.07	6.60	13.30	3.6
	*qPLHT5*.*1*	2016–17	CaLG05	AX-123653052	AX-123631649	1.07	7.62	3.50	8.70	2.4
	*qPLHT8*.*1*	2016–17	CaLG08	AX-123638424	AX-123657820	13.74	14.30	3.60	8.10	2.2
Number of primary branches (PB)	*qPB*.*2*.*1*	2015–16	CaLG02	AX-123621195	AX-123621154	111.10	111.40	3.40	8.70	-0.1
	*qPB*.*3*.*1*	2015–16	CaLG03	AX-123622729	AX-123622639	14.30	14.40	6.50	59.70	-0.3

*LOD*, logarithm of odds; *PVE*, phenotypic variance explained; *cM*, centimorgan.

Furthermore, if a QTL for a particular trait was identified for more than one year, it was considered a consistent QTL, as described previously [19]. Accordingly, one consistent QTL for PLHT (*qPLHT4*.*1*) was identified on CaLG04 (Table 3). When a particular marker was found to flank more than one QTL, that particular region was considered a single genomic region. The sequences and physical positions of the markers flanking the identified QTLs are provided in the S2 Table.

#### QTLs for yield and yield component traits

*Yield per plant (YPP)*. Two major QTLs, one each on CaLG04 (*qYPP4*.*1*) and CaLG01 (*qYPP1*.*1*), explaining 10.1% PVE (2015–16) and 36.2% (2016–17), respectively, were identified (Table 3).

*100-seed weight (100SW)*. A total of 6 QTLs were identified for 100SW across three years. In 2015–16, one major QTL on CaLG06 (*q100SW6*.*1*) explaining 13% PVE, and one minor QTL on CaLG07 (*q100SW7*.*1*; 8.1% PVE), were identified. In 2016–17, one major QTL on CaLG06 (*q100SW6*.*2*) that explained 11.3% PVE, and two minor QTLs, one each on CaLG03 (*q100SW3*.*1*; 6.5% PVE) and CaLG07 (*q100SW7*.*2*; 7.8% PVE), were identified. In 2017–18, only one major QTL was identified on CaLG04 (*q100SW4*.*1*), which explained 16.6% PVE. Interestingly, this QTL was also found to overlap with a consistent QTL identified for plant height (*qPLHT4*.*1*) (Table 3).

*Pods per plant (PPP)*. Analysis of PPP data led to the identification of only one major-effect QTL on CaLG06 (*qPPP6*.*1*), explaining 15.2% PVE for 2015–16 (Table 3).

#### QTLs for agronomic traits

*Plant height (PLHT)*. One consistent major-effect QTL was identified on CaLG04 (*qPLHT4*.*1*), explaining 18.5% and 13.3% PVE in 2015–16 and 2016–17, respectively. In addition, one major-effect QTL on CaLG01 (*qPLHT1*.*1*) explaining 14.2% PVE was identified for 2016–17. Furthermore, two minor-effect QTLs, one each on CaLG05 (*qPLHT5*.*1*; 8.7% PVE) and on CaLG08 (*qPLHT8*.*1*; 8.1% PVE), were also identified for 2016–17 (Table 3).

*Number of primary branches (PB)*. Two QTLs for PB trait were detected for 2015–16. These include one major-effect QTL on CaLG03 (*qPB3*.*1*), explaining 59.7% PVE, and one minor-effect QTL on CaLG02 (*qPB2*.*1*), explaining 8.7% PVE (Table 3).

### Analysis of epistatic interactions

The combinations of two- and three-loci interactions were scrutinized for all the studied traits across three years. Epistatic QTLs (E-QTLs) were identified only for 100SW evaluated during the years 2015–16 (S3 Table), and 2016–17 (S4 Table). Using the two-loci interaction model, a total of 95 E-QTLs were identified each for 2015–16 and 2016–17. The majority of these interactions were observed between CaLG04 and CaLG06. For instance, two-loci interactions between AX-123632830(AA) and AX-123624139(AA) explained 62.48% PVE during 2015–16, and 64.16% PVE during 2016–17. In addition, a total of 356 E-QTLs were detected for 2016–17, using the three-loci interaction model. These E-QTLs were also mainly detected between CaLG04 and CaLG06 and explained about 64.15–65.55% PVE observed for this mapping population. For instance, three-loci interactions between the alleles AX-123632830(AA), AX-123640164(AA) and AX-123624139(AA) explained about 65.55% PVE.

### Candidate genes underlying the QTLs detected for 100-seed weight and plant height

The genes underlying the marker intervals of 6 QTLs for 100SW and 4 unique QTLs for PLHT were retrieved, and a total of 1,476 genes were detected (S5 and S6 Tables). The 6 QTLs detected for 100SW were found to harbour 417 genes (S5 Table). Based on the gene ontology (GO) annotation, out of 417 genes, 11 key genes were prioritized based on their role in controlling seed parameters in chickpea and other crops. These include one gene on CaLG03 [pentatricopeptide repeat-containing protein At2g03880 (*Ca_06269*)], 5 genes on CaLG04 [tubby-like F-box protein 8 (*Ca_04384*), pentatricopeptide repeat-containing protein At5g52850 (*Ca_04422*), pentatricopeptide repeat-containing protein At1g55630-like (*Ca_04472*), homoserine kinase (*Ca_04479*), transcription factor bHLH118 (*Ca_04526*)], and 5 genes on CaLG06 [ethylene-responsive transcription factor ERF043-like (*Ca_15786*), bidirectional sugar transporter SWEET2-like (*Ca_23961*), zinc finger, C3HC4 type (RING finger) protein (*Ca_19553*), cytochrome P450 83B1-like (*Ca_15917*), cytochrome P450 72A15-like (*Ca_19813*)].

A total of 1059 genes were found underlying the four QTL regions detected for PLHT. Out of these 1059 genes, 13 putative genes (two on CaLG01, five on CaLG04 and six on CaLG05) were prioritized based on their role in regulating plant height in crop plants (S6 Table). These included type-II homeodomain-leucine zipper protein (*Ca_21404*), and basic-leucine zipper transcription factor I (*Ca_19454*) on CaLG01; ethylene-responsive transcription factor ERF110-like isoform (*Ca_04370*), protein IQ-DOMAIN 14-like (*Ca_04493*), ethylene responsive transcription factor ERF027-like (*Ca_04503*), cytochrome P450 704C1-like isoform (*Ca_04534*), and C2H2-like zinc finger protein (*Ca_04451*) on CaLG04; probable basic-leucine transcription factor I (*Ca_18221*), NADH ubiquinone oxidoreductase (*Ca_20087*), NADH dehydrogenase [ubiquinone] 1-alpha subcomplex assembly factor 2 (*Ca_20086*), cytochrome c biogenesis C (*Ca_20073*), cytochrome c biogenesis C (*Ca_20070*), and NAD(P)H-quinone oxidoreductase subunit N (*Ca_08896*) on CaLG05.

## Discussion

Breeding for yield, yield components and agronomic traits could enhance crop productivity. Recent developments in plant genomics have led to new and improved breeding methodologies, which have vastly accelerated the breeding process [27–29]. These advances have facilitated detecting genomic regions/QTLs linked with desirable traits and, thereby, identification of genes controlling both simple and complex traits [20, 24, 30, 31]. Trait linked markers have facilitated marker-assisted breeding [13, 19, 32] and positional cloning for different traits in crop plants [4, 26, 33, 34]. Detection of polymorphism in chickpea has been a major constraint due to the limited genetic diversity [15, 35]. Such low levels of genetic diversity in the cultivated gene pool entailed the need for targeting the inter-specific polymorphisms between wild and cultivated chickpea accessions [36, 37]. Recent advances in genome-based capabilities have enabled the development of high-throughput approaches for genotyping, allowing the detection of desirable alleles and multiple QTLs having the potential to affect desired responses. Therefore, the current study aimed to utilize the high-density genetic map of ICC 4958 × DCP 92–3 RIL population for detecting main-effect QTLs for yield, yield components, and agronomic traits; and aimed to identify potential candidate genes for 100SW and PLHT.

Detailed analysis of phenotyping data revealed a substantial genotypic variation among the RILs for all studied traits across three years. In the present study, the extent of genotypic variances was more than their corresponding environmental variances for PLHT, PPP, 100SW and YPP, indicating a greater contribution of the genotypic component to the total variation in these traits [19, 38–40]. All traits, except 100SW, displayed transgressive segregation in both directions, suggesting that both parental lines contributed favorable alleles for these traits. For 100SW, transgressive segregation, mostly in a negative direction, was observed for multiple RILs across all three years, which might be because of unwanted linkages between desirable and undesirable alleles contributed by parental lines. Combined correlation analysis results displayed a high degree of correlation for 100SW between years, which is predicted to be due to the high heritability of this trait observed in chickpea [19]. Furthermore, PLHT displayed a moderate correlation between 2015–16 and 2016–17, and between 2016–17 and 2017–18. However, all remaining traits displayed a low degree of correlation between years, which may be due to the significant influence of the genotypes’ environment. Significant genotypic and environmental differences and a low correlation observed for traits between years might be the reasons for the absence of any consistent QTL detected for the evaluated traits.

In the present study, the genotyping of RILs using a high-throughput Axiom^®^*CicerSNP* array facilitated the construction of a dense genetic map. The genetic map comprising of 3,818 SNPs, with an average inter-marker distance of 0.30 cM and an average density of 3.91 SNPs/cM, is one of the most saturated maps developed for chickpea, which is superior to some of the previously reported inter-specific maps [9, 36, 41, 42]. Furthermore, a total of 16 QTLs were mapped for five traits, with PVE ranging from 6.5% to 59.70%. Here, two major-effect QTLs (*qYPP4*.*1* and *qYPP1*.*1*) for yield per plant were identified for 2015–16 and 2016–17, with PVE ranging from 10.10 to 36.20%. In contrast to earlier studies [19, 43, 44], the major-effect QTL *qYPP1*.*1* explained the highest phenotypic variance of 36.20% and may hold potential for deployment in chickpea breeding efforts by marker-assisted breeding approach. Despite high PVE values obtained for yield per plant and the number of primary branches QTLs, only a marginal additive effect was observed for these traits across different years. This suggested the absence of any significant difference in trait values between parents. The high phenotypic variation explained by QTLs for YPP and PB may be due to the sample size of the mapping population (N = 161), density of markers used in the linkage map, and QTL mapping software. Recent QTL mapping studies suggest epistasis to be a crucial genetic component underlying complex quantitative traits such as 100-seed weight, plant height, yield and components [19]. Importantly, the use of epistatic effects in marker-assisted selection holds the potential to achieve a higher genetic gain in breeding programs. In the present study, two-loci and three-loci epistatic QTL interactions were identified for 100-seed weight across two years. Inclusion of these epistatic effects in sophisticated biological models will provide an opportunity to optimize long-term selection response and a comprehensive understanding of the genetic base underlying improvement of 100-seed weight. Moreover, deployment of the identified epistatic QTLs will enable marker-assisted selection to bear a longer persistence response and may lead to a considerable increase in genetic gain.

Yield trait is complex and governed by several components such as pod weight, haulm weight, harvest index, seed weight, pod to flower ratio, etc., and QTLs associated with these traits are favourable targets for selection. Furthermore, plant height represents a crucial factor for machine harvest because the losses incurred during machine harvest is more for semi-erect genotypes (about 20%) and less in tall and erect genotypes (2.6–5.0%) [45]. Given the shortage in the workforce and to address drudgery among farmers, there is a huge demand for tall and erect varieties suitable for machine harvesting. A total of 6 QTLs for 100SW were identified across three years, including two QTLs each on CaLG06 and CaLG07, and one QTL each on CaLG03 and CaLG04, explaining phenotypic variation ranging from 6.50% to 16.60%. Many studies have reported QTLs for 100SW in different genetic backgrounds of chickpea in the last few years [10, 46–48]. For example, the QTLs for 100SW were identified on CaLG01 and CaLG04 and accounted for 37% of the phenotypic variance across two environments [10]. QTLs for seed weight have also been reported on CaLG02 and CaLG05 in different genetic backgrounds [9, 43, 48–52]. Many QTLs identified in the present study were found in close proximity or overlapped earlier reported QTLs. For instance, *q100SW4*.*1* on CaLG04 was detected in close proximity of two QTLs consistent across years and locations reported earlier [19, 53], but in a different mapping population. However, higher PVE and LOD scores of the QTLs identified in the same region for previous studies compared to the present study could be due to the difference in the RIL population used and genotype × environmental interaction effects. The other two major-effect QTLs, including *q100SW6*.*1* and *q100SW6*.*2* on CaLG06, reported in this study are novel QTLs. Similarly, for plant height, three major-effect and two minor-effect QTLs with PVE ranging from 8.1% to 18.5% were identified. When flanking markers of the QTLs identified for plant height in the present study were compared with the markers for plant height QTLs identified in previous studies [7, 19, 40, 54, 55], it was observed that all the QTLs identified in the present study were novel and did not exhibit any similarity with previously reported QTLs. Hence, novelty and population-specific characteristics of the identified QTLs governing plant height was observed in the present study.

Identifying candidate genes for a particular trait is the first important step to understand the molecular mechanisms underlying the trait of interest. Integration of genomics-based knowledge with conventional breeding efforts can decipher molecular mechanisms underlying traits of interest. With the availability of the chickpea genome sequence [1], it is now possible to identify genes governing traits like seed weight and plant height [26]. Genomic regions harboring QTLs for 100SW and PLHT were selected to identify putative candidate genes controlling these traits. Based on these premises, a total of 417 genes were found underlying 6 QTLs detected for the 100SW, and several genes within this set were shown to play a significant role in seed development in previous studies. For instance, a gene encoding C3HC4-type RING finger protein (*Ca_19553*) was shown to control plant growth and fruit development in *Nicotiana benthamiana* [56]. Genes including serine kinase (*Ca_04479*) and pentatricopeptide repeat containing proteins (PPR) (*Ca_06269*, *Ca_04422*, *Ca_04472*) have been shown to play a major role in seed development in different crops [57–59]. A sugar transporter protein encoding gene (*Ca_23961*) was also displayed to play a key role in the accumulation of seed reserve and transport in wheat [60], *Arabidopsis* [61], and fava bean [62]. Furthermore, tubby-like F-box protein 8 (*Ca_04384*), transcription factor bHLH-118 (*Ca_04526*), RING-H2 finger protein (*Ca_19553*), and ethylene-responsive factor ERF (*Ca_04503*) displayed high expression in seed tissue, thereby predicting their role in regulating seed development in *Arabidopsis* [63] and chickpea [46].

We also predicted the possible candidate genes underlying four unique QTLs for PLHT. The potential gene underlying the *qPLHT1*.*1* genomic region includes a leucine zipper protein (*Ca_21404*). Moreover, the most promising genes underlying *qPLHT5*.*1* genomic region on CaLG05, include NADH dehydrogenase (*Ca_20086*), and cytochrome-c biogenesis (*Ca_20070*, *Ca_20073*). Integrated genomic approaches elucidated the role of these genes in regulating plant height. These genes are known to play a key role in the tricarboxylic acid (TCA) cycle and electron transport chain (ETC) for regulating respiration and mitochondrial organization in crop plants [64–67]. Another gene family underlying the *qPLHT5*.*1* genomic region on CaLG05 includes the *bZIP* transcription factor genes, which regulate plant morphology using gibberellins (GAs) and GA homeostasis. It was shown that down-regulation of genes involved in GA biosynthesis inhibits cell elongation and growth of stem internodes and results in dwarf phenotype in monocot and dicot crop plants [68]. GA response modulators such as dwarf 8 (*d8)*, semi-dwarf (*Sd1)*, reduced height (*Rht*) and gibberellin insensitive (*GAI*) that regulate plant height have been identified and validated in several crops, including wheat, maize, rice and tobacco [68–71]. The interaction and similar expression characteristics of these genes in a regulatory pathway underlying both mitochondrial respiration and GA biosynthesis are necessary for maintaining the growth and development of organs, including plant height [72, 73].

## Materials and methods

### Development of RIL population

A RIL population was developed by crossing ICC 4958 (large seeded and drought tolerant donor parent) with DCP 92–3 (small seeded and drought susceptible) chickpea genotype at ICAR-IIPR. The segregants displayed variations in traits such as yield, 100-seed weight and plant height. This mapping population containing 166 RILs (F_8_) was developed at ICAR-Indian Institute of Pulses Research (IIPR), Kanpur, India. The population was advanced by the single seed descent (SSD) method to develop recombinant inbred lines.

### Phenotypic evaluation of yield, yield components and agronomic traits

A total of 166 RILs were evaluated during the Rabi season for three years (2015–16, 2016–17 and 2017–18) at ICAR-IIPR, Kanpur (26° 20’ 22.884’’ N80° 17’ 34.584’’ E), India. The RIL population was sown in a plot size of 1.2 m^2^ (4 m × 3 m), and seed to seed distance of 10 cm was maintained in a row. The field was prepared for sowing by applying diammonium phosphate (18% N and 46% P_2_O_5_). During the crop season, rainfall ranged from 196 to 230 mm, and surface irrigation was applied during the vegetative stage as and when required. The experiment was conducted in augmented design. The traits that were evaluated during the experiment include yield per plant (YPP), 100-seed weight (100SW), pods per plant (PPP), plant height (PLHT), number of primary branches (PB), and number of secondary branches (SB). Three plants were randomly selected from each plot, and the phenotypic data for the traits mentioned above was recorded. The mean value of the data recorded on three plants was computed and used for further analysis.

### Genotyping of RILs

Genomic DNA was extracted from parents and RILs using a modified CTAB method as described previously [74]. In brief, young leaves of 20–25 days old plants were used for DNA isolation, and DNA quality was tested and quantified using NanoDrop 8000 spectrophotometer (Thermo Scientific, USA). DNA concentration was normalized to a minimum of 40 ng/μL. Based on the presence of high-quality total genomic DNA, 161 RILs were genotyped using the Axiom^®^*CicerSNP* array containing 50,590 SNPs distributed across eight linkage groups of chickpea as described earlier [4].

### Genetic map construction and QTL analysis

The genotyping data obtained from the Axiom^®^*CicerSNP* array was utilised for the construction of a genetic map. SNPs indicating the presence of contrasting alleles between two parents were targeted. For the 17,173 polymorphic SNPs thus obtained, a chi-square test was carried out with a null hypothesis that two alleles at a given locus segregate in a 1:1 ratio for a RIL population. SNP markers that showed substantial deviation from the 1:1 ratio, and high missing data were not used for further analyses. A high-density genetic map for ICC 4958 × DCP 92–3 RIL population was constructed using JoinMap v4.1 [75]. The logarithm of odds (LOD) score for the test of linkages between marker pairs was set to 3.0, and the markers that were attributed to a linkage group at a LOD grouping threshold of 3.0 were utilized. The maximum-likelihood mapping algorithm and Kosambi mapping function were used for constructing the genetic map. In order to map QTLs associated with yield, yield components and agronomic traits, the field phenotyping data for 161 RILs collected across three years was used. QTL mapping was performed using Windows QTL Cartographer software version 2.5. Here, composite interval mapping using a genome-wide LOD threshold of 3.0 was performed at *p*<0.05 significance, as described previously [47, 76, 77]. This led to the mapping of main-effect and minor-effect QTLs associated with the traits evaluated in this mapping population.

### Mining of candidate genes

To retrieve candidate genes underlying the QTL intervals of 100-seed weight and plant height, the QTL flanking markers were selected and used for similarity search against chickpea reference genome assembly (CaGAv1.0) [1] using blastn (with the parameter “-task blastn-short”). The flanking markers were then used to identify genes (between left and right markers) using bedtools (v2.17.0) against the corresponding chickpea genome, and 1,476 genes in the QTL regions were retrieved. Furthermore, the identified genes were functionally annotated using blastx (E-value cutoff 1E-05) against the NCBI nr database, followed by GO annotation using Blast2GO (v5.2).

### Statistical analyses

Phenotyping data measured across three years were analysed individually to estimate the best linear unbiased predictors (BLUPs) for each trait using the REML option in the PROC MIXED procedure of SAS v9.0 (SAS Institute Inc., NC, USA). The performance of a genotype for augmented randomized complete block design was modelled as:

$$Y_{ij}=\mu +\beta_{i}+c_{j}+\alpha_{i}+\varepsilon_{ij}$$

where *Y*_*ij*_ is the phenotype of the i^th^ genotype in the j^th^ block, *μ* is the overall mean, *ß*_*i*_ is the block effect which was considered as random, *c*_*j*_ is the effect of the check in j^th^ block which was considered as fixed, *α*_*i*_ is the random effect of the i^th^ genotype, and *ε*_*ij*_ is the residual considered as a random effect. The phenotyping data collected across three years were subjected to ANOVA for RCBD using the ‘augmentedRCBD’ package in R [78]. The phenotypic variation observed for each of the six traits was evaluated using the formulae described previously [79, 80]:

$$Coefficient\;of\;variation\;(\%CV)=\frac{\sqrt{MS_{g}}}{\bar{X}}\times 100$$

$$Phenotypic\;coefficient\;of\;variation\;(PCV)=\frac{\sqrt{\sigma_{p}^{2}}}{\bar{X}}\times 100$$

$$Environmental\;coefficient\;of\;variation\;(ECV)=\frac{\sqrt{\sigma_{e}^{2}}}{\bar{X}}\times 100$$

where $\bar{X}$ indicates the grand mean for each trait.

A Pearson correlation analysis and principal component analysis was computed using a custom R script. The frequency distribution of the yield, yield components, and agronomic traits within the RIL mapping population was analysed and plotted with the ‘UsingR’ package in R. The analysis of epistatic interactions between the interacting QTLs was conducted using Genotype Matrix Mapping (GMM) software (version 2.1; http://www.kazusa.or.jp/GMM) [81]. For estimating the combinations of two-loci and three-loci interactions using the GMM algorithm, the maximum length of locus combinations was set to 2 and 3, respectively. Moreover, the minimum number of corresponding samples was set to 1, and the default option of ‘automatic’ was used for defining the search range (d). Here, the syntax ‘AA’ refers to ICC 4958 alleles, while the syntax ‘BB’ refers to DCP 92–3 alleles.

## Conclusion

In the present study, analysis of the genotyping data generated on ICC 4958 × DCP 92–3 RIL mapping population using Axiom^®^*CicerSNP* genotyping array facilitated the construction of a high-density genetic linkage map. Analysis of the phenotyping data for six traits evaluated across three years along with the genotyping data led to the identification of 16 major- and minor-effect QTLs for five traits, explaining up to 59.7% PVE. Genes underlying the identified QTL regions for 100SW and PLHT were reported to play a key role in regulating seed attributes, plant height, and plant growth and development. However, further fine-mapping and experimental validation of these genes is needed to precisely determine the candidate gene(s) underlying the QTLs identified. Nonetheless, the high-density linkage map, major-effect QTLs and genomic regions identified in this study hold huge potential to be deployed in chickpea improvement programs by the marker-assisted breeding approach to develop high yielding chickpea varieties.

## Supporting information

S1 Table Descriptive statistics and analysis of variance for six traits evaluated across three years in ICC 4958 × DCP 92–3 RIL population. (XLSX)

S2 Table Name, physical position and sequence information for markers flanking the identified QTLs. (XLSX)

S3 Table List of epistatic QTLs (E-QTLs) identified for 100-seed weight measured during 2015–16. (XLSX)

S4 Table List of epistatic QTLs (E-QTLs) identified for 100-seed weight measured during 2016–17. (XLSX)

S5 Table List of genes underlying the major-effect QTL regions identified for 100-seed weight. (XLSX)

S6 Table List of genes underlying the major-effect QTL regions identified for plant height. (XLSX)

S1 Fig Frequency distribution of yield, yield components and agronomic traits evaluated during 2015–16. Frequency distribution for (a) yield per plant (YPP_2015–16), (b) 100-seed weight (100SW_2015–16), (c) pods per plant (PPP_2015–16), (d) plant height (PLHT_2015–16), (e) number of primary branches (PB_2015–16) and (f) number of secondary branches (SB_2015–16). Red arrow indicates the trait value for ICC 4958 and blue arrow indicates the trait value for DCP 92–3.
(TIF)

S2 Fig Frequency distribution of yield, yield components and agronomic traits evaluated during 2016–17. Frequency distribution for (a) yield per plant (YPP_2016–17), (b) 100-seed weight (100SW_2016–17), (c) pods per plant (PPP_2016–17), (d) plant height (PLHT_2016–17), (e) number of primary branches (PB_2016–17) and (f) number of secondary branches (SB_2016–17). Red arrow indicates the trait value for ICC 4958 and blue arrow indicates the trait value for DCP 92–3.
(TIF)

S3 Fig Frequency distribution of yield, yield components and agronomic traits evaluated during 2017–18. Frequency distribution for (a) yield per plant (YPP_2017–18), (b) 100-seed weight (100SW_2017–18), (c) pods per plant (PPP_2017–18), (d) plant height (PLHT_2017–18), (e) number of primary branches (PB_2017–18) and (f) number of secondary branches (SB_2017–18). Red arrow indicates the trait value for ICC 4958 and blue arrow indicates the trait value for DCP 92–3.
(TIF)

S4 Fig Principal component analysis for yield, yield components and agronomic traits evaluated across three years. Principal component analysis for the years (a) 2015–16, (b) 2016–17 and (c) 2017–18 in the ICC 4958 × DCP 92–3 RIL population. In the PCA biplot, colored dots represent diverse RILs and their position on the plot is relative to specific trait loadings corresponding to PC1 and PC2. Also, positively correlated variables are clustered together, while variables that are negatively related are placed on the opposite side of the origin. YPP, yield per plant; 100SW, 100-seed weight; PPP, pods per plant; PLHT, plant height; PB, number of primary branches; SB, number of secondary branches.
(TIF)

S5 Fig Correlation heat-maps for yield, yield components and agronomic traits. Pearson correlation analysis heat-maps for six traits evaluated across three years (a) 2015–16, (b) 2016–17 and (c) 2017–18 in the ICC 4958 × DCP 92–3 RIL population. YPP, yield per plant; 100SW, 100-seed weight; PPP, pods per plant; PLHT, plant height; PB, number of primary branches; SB, number of secondary branches.
(TIF)
