# Supplementary figures and images for: Construction of a high-density genetic map and QTL analysis for yield, yield components and agronomic traits in chickpea (Cicer arietinum L.)
Source: PLoS One. 2021 May 14;16(5):e0251669. doi: 10.1371/journal.pone.0251669 (PMC8121343; doi:10.1371/journal.pone.0251669)

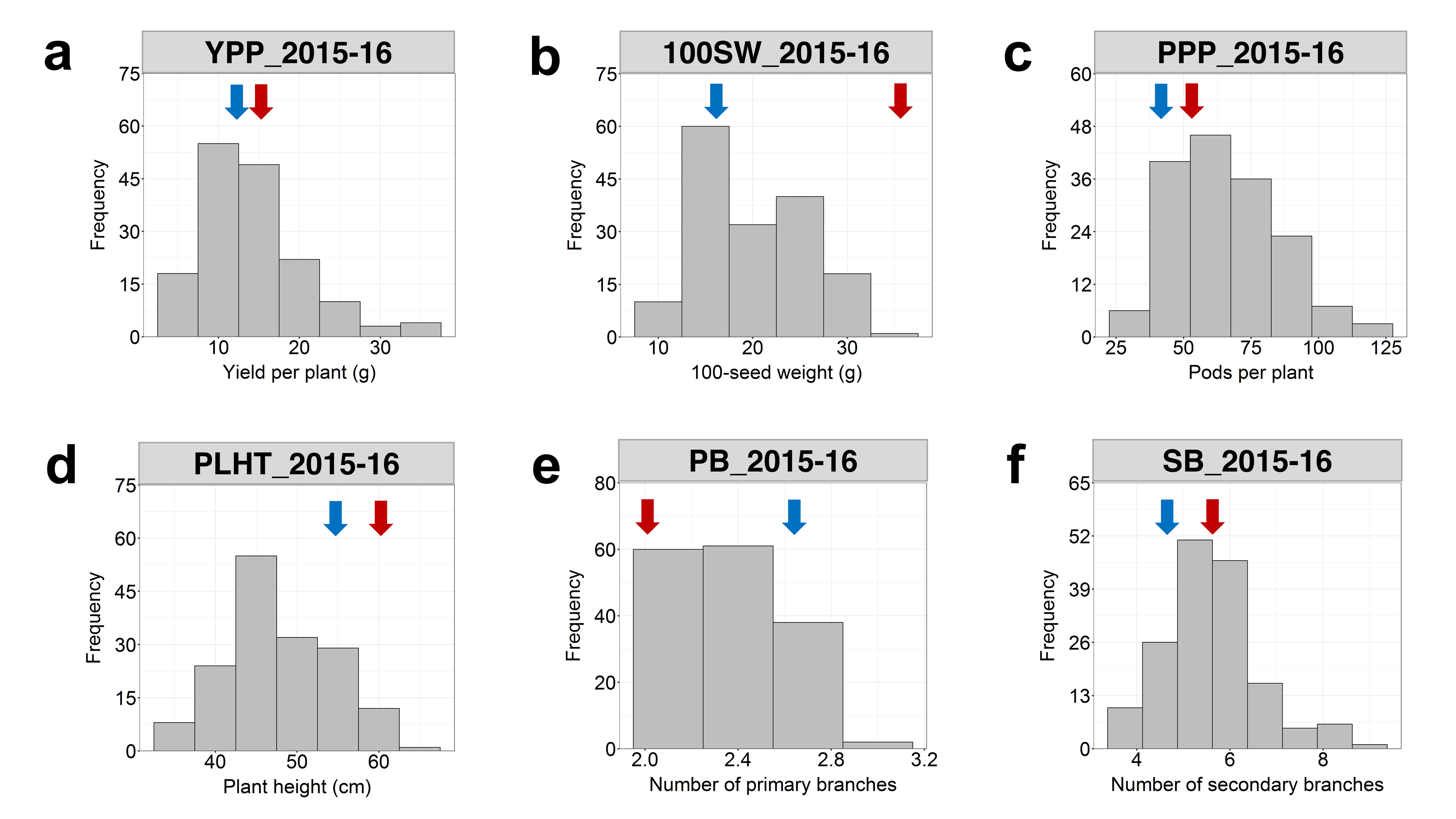

Supplement: S1 Fig — Frequency distribution for (a) yield per plant (YPP_2015–16), (b) 100-seed weight (100SW_2015–16), (c) pods per plant (PPP_2015–16), (d) plant height (PLHT_2015–16), (e) number of primary branches (PB_2015–16) and (f) number of secondary branches (SB_2015–16). Red arrow indicates the trait value for ICC 4958 and blue arrow indicates the trait value for DCP 92–3. (TIF) [file pone.0251669.s007.tif]

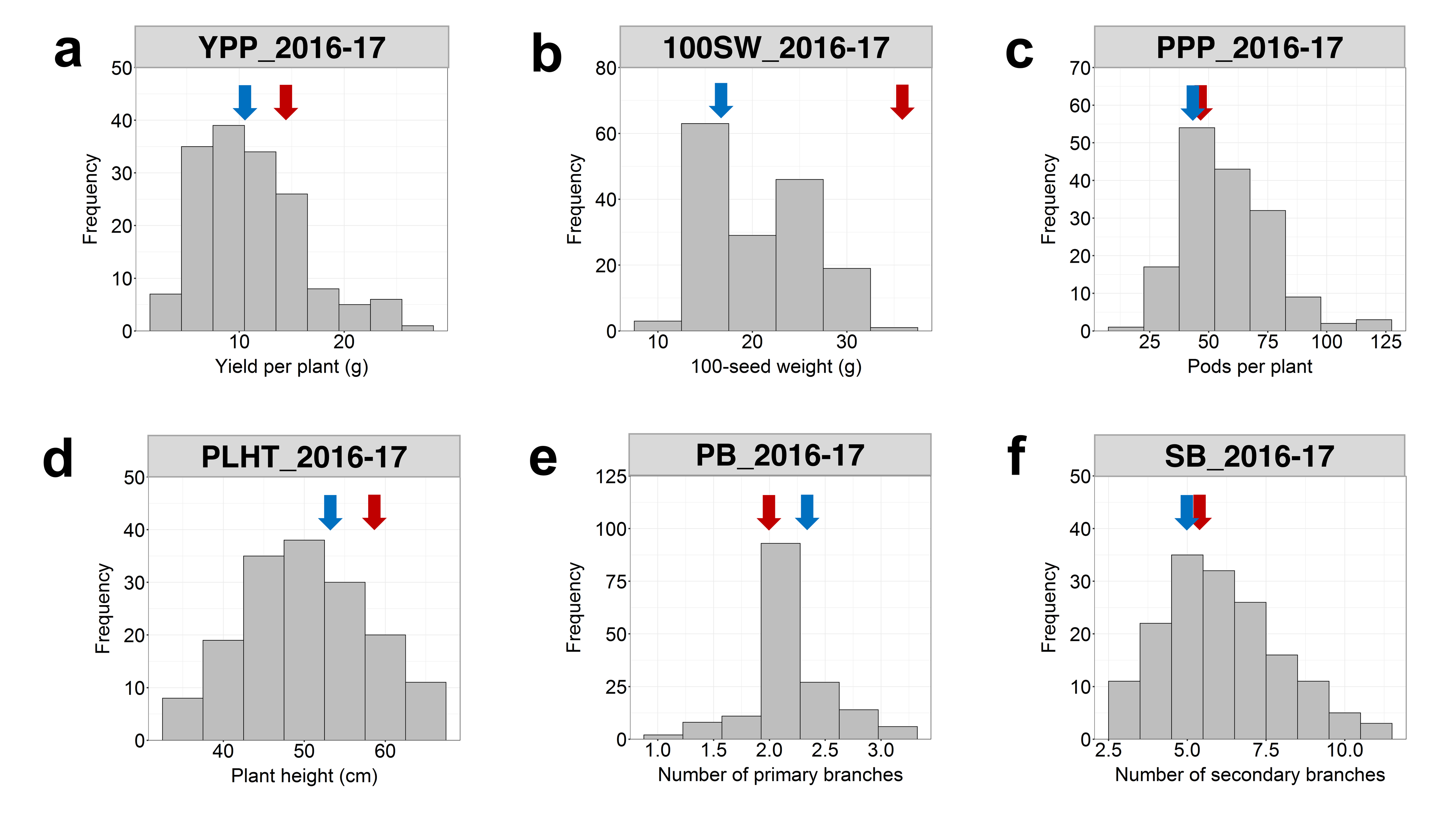

Supplement: S2 Fig — Frequency distribution for (a) yield per plant (YPP_2016–17), (b) 100-seed weight (100SW_2016–17), (c) pods per plant (PPP_2016–17), (d) plant height (PLHT_2016–17), (e) number of primary branches (PB_2016–17) and (f) number of secondary branches (SB_2016–17). Red arrow indicates the trait value for ICC 4958 and blue arrow indicates the trait value for DCP 92–3. (TIF) [file pone.0251669.s008.tif]

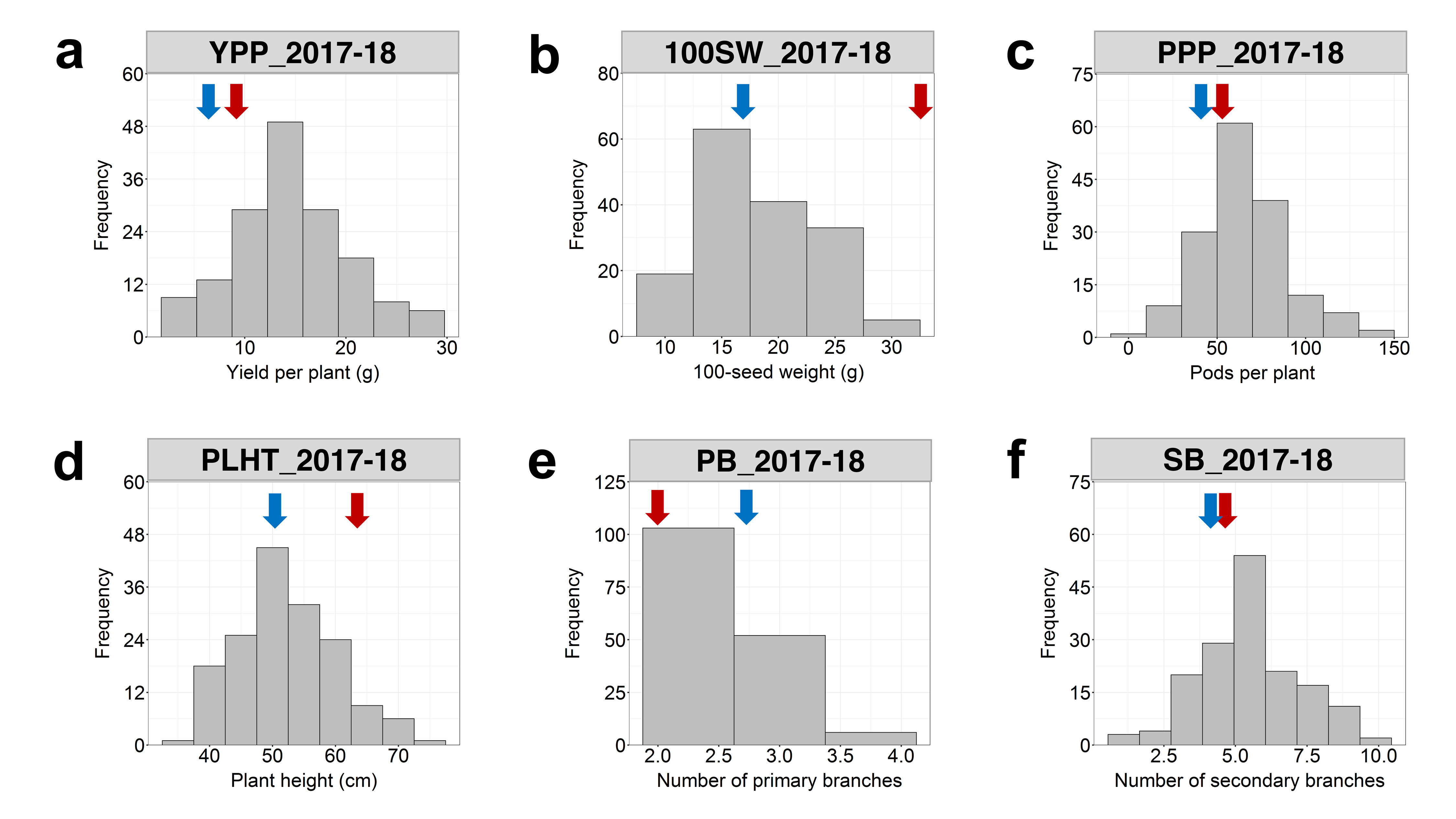

Supplement: S3 Fig — Frequency distribution for (a) yield per plant (YPP_2017–18), (b) 100-seed weight (100SW_2017–18), (c) pods per plant (PPP_2017–18), (d) plant height (PLHT_2017–18), (e) number of primary branches (PB_2017–18) and (f) number of secondary branches (SB_2017–18). Red arrow indicates the trait value for ICC 4958 and blue arrow indicates the trait value for DCP 92–3. (TIF) [file pone.0251669.s009.tif]

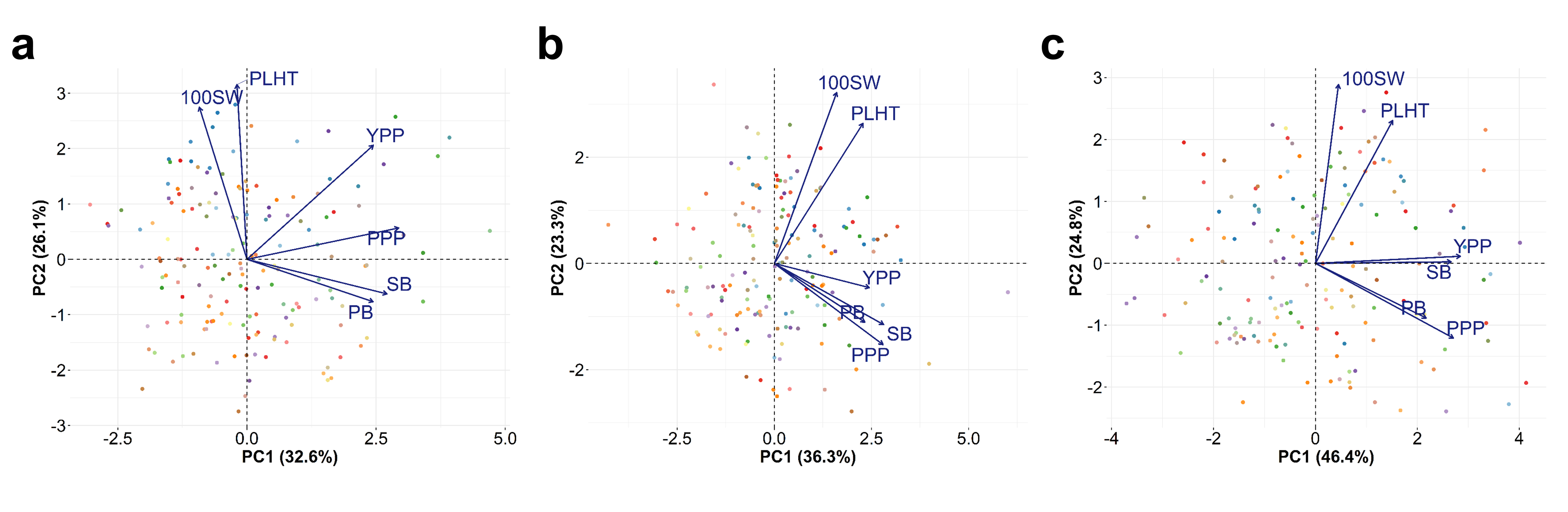

Supplement: S4 Fig — Principal component analysis for the years (a) 2015–16, (b) 2016–17 and (c) 2017–18 in the ICC 4958 × DCP 92–3 RIL population. In the PCA biplot, colored dots represent diverse RILs and their position on the plot is relative to specific trait loadings corresponding to PC1 and PC2. Also, positively correlated variables are clustered together, while variables that are negatively related are placed on the opposite side of the origin. YPP, yield per plant; 100SW, 100-seed weight; PPP, pods per plant; PLHT, plant height; PB, number of primary branches; SB, number of secondary branches. (TIF) [file pone.0251669.s010.tif]

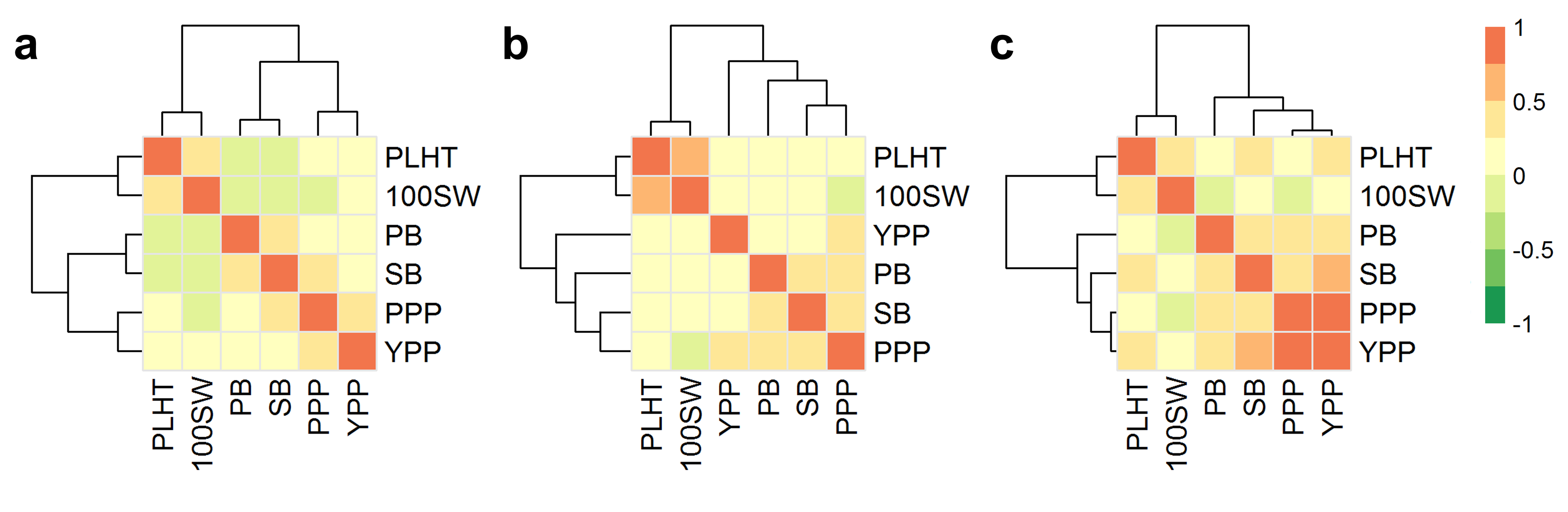

Supplement: S5 Fig — Pearson correlation analysis heat-maps for six traits evaluated across three years (a) 2015–16, (b) 2016–17 and (c) 2017–18 in the ICC 4958 × DCP 92–3 RIL population. YPP, yield per plant; 100SW, 100-seed weight; PPP, pods per plant; PLHT, plant height; PB, number of primary branches; SB, number of secondary branches. (TIF) [file pone.0251669.s011.tif]
